# Monthly Periscope

**Published:** 1854-04

**Authors:** 


*Monthly Periscope.*— Our Journal of last month contained no Periscope. This omission was rendered necessary by the amount of editorial matter which required timely attention. And, as it happens, we have this month so much original matter, that the Periscope only finds entrance by the omission of the Eclectic Department.

We look on our selected matter as, usually, not the least important portion of our pages. But as it is our purpose to publish, monthly, original matter which may be interesting to our circle of readers, we have usually curtailed our selections, and in lieu of large borrowing from the pages of our exchanges, we have given this our Periscope — a department in which we gather together many scattered facts of value, for which we would have no room were we to publish them in their original form. This method gives us also the opportunity of criticism, in the conduct of which we strive to be liberal, but, at the same time, we limit it by no rules, and dwell as little, or as long, as we please on any given subject.

*Meigs on the Uterus.*— The "American Medical Monthly," the new Journal in New York, has a very "square-toed" review of Meigs on the Uterus, as constituting a part of the transactions of the American Medical Association. In our own notice of this work, (in preceding pages) we have looked upon the report as a book; while the "Monthly" looks upon the book as a report. This is a somewhat important difference. In the form in which we recognize it, it is a very fair tract upon its special subject; hardly up to the reputation of the writer, perhaps, but nevertheless a very good and useful book. But as a *report*, we agree with the "Monthly," that a clinical report from Dr. Meigs, of cases however interesting, does not constitute a report of the existing condition of obstetrical science for the year of our Lord 1853.

*New Entozoon.*— Dr. S. Mitchell gives in the Boston Medical and Surgical Journal, a description of a new inhabitant of the intestinal canal, of which over one hundred were expelled from a little girl of five years, by the pumpkin-seed emulsion. Deprive a young unfledged bird of wings and legs, and let a long, spindling tail grow from each extremity, the anterior of which shall be fimbriated, the whole animal being some inch and a half or two inches long, and you have as good a description of the thing as we can give without a wood cut.

*Vegetable growth in the "fur" of a coated tongue.*— Prof. Alonzo Clark presented to the New York Pathological Society, the results of some recent inquiries made by him, into the nature of the "fur" found in large quantities on the tongue of a patient in a very low condition.

The fur had "a mossy white appearance, and was even ⅛ of an inch thick, or more, covering the mucous membrane of the lips and cheeks, as well as the tongue."

It was found, under the microscope, to consist of

1st. Epithelial scales.

2d. Vibriones, and

3d. An abundant vegetable growth.

In the scales there was nothing remarkable. The vibriones were very large and active animalculæ. The spores from which the vegetable fibers were produced, were "sometimes imbedded in the epithelial cells, and sometimes packed between them"—Its quantity was astonishingly great. Dr. Clark found, after research, that he had been preceded by M. Robin a few months in this discovery. This growth is probably common in many exhausting diseases. It is believed to be constantly present in the disease called "miguet" by the French. The sulphurous acid is found to destroy this parasitic vegetation, and may be considered the most appropriate remedy for it.

*Arabian Medicine.*— On the second day of the late session of the New York State Medical Society, Dr. Van Dyck, an honorary member, gave a very interesting account of the state of medicine in Syria. Dr. Van Dyck has for some time been attached to the Mission Establishment at Smyrna.

The condition of medical science in the East is much the same as it was in the days of Rhazes and the Abn Sina, (corrupted into Avicenna by the moderns.) Indeed, the old authors of primitive medicine are still in repute, and, like the Dogmatists, who ruled the profession in the middle ages, they look to the ancients as not only the first, but the highest authority ; regarding them in the same light as they do the Koran, and rarely departing from their precepts.

Their surgery is very simple. In bleeding they feel for the *artery* at the bend of the arm, not with the view of avoiding, but of penetrating it. They evidently do not understand the distinction between arteries and veins, as they ligate the arm above the point of venesection, and always select the point where the vein crosses the artery. Of course they only reach the vein.

That they do not wound the artery is not very surprising to any one who has ever taken the pains to notice how often venesection is performed in this country, directly over the artery. It is a most common thing to feel the pulsation of the brachial immediately beneath the cicatrices of old venesections. That much carelessness is not oftener attended by dangerous consequences, is the only remarkable circumstance. The instrument used by the Arabs, is oftentimes only a sharp piece of glass, attached to a stick, which is placed over the vessel and gently struck, as in the use of the phleam in bleeding animals.

Dr. Van Dyck mentions a man, Ahu Budka, who performs lithotomy with a single knife. We have elsewhere heard an account of his method, which is to introduce his finger into the rectum, and after finding the stone, to work his finger round it, (the vesico-rectal septum of course intervening,) and drag it down against the perineum. The incision is thus made directly upon it, and when completed, the pressure of the finger behind throws out the stone.

The operation has the merit of simplicity, and is said to afford a fair average of success.

*Uses of Strychnia.*— Perusing a reference to Dr. Corson's late paper on functional disease of the heart, we noticed an allusion to his use of strychnia in debility of the heart.

This drug is but little in use among the generality of practitioners. Probably its active poisonous nature has deterred them; yet it is no more dangerous in its use than many other drugs which we employ without hesitation. Early in our practice we prescribed the solution of strychnia in acetic acid, in a number of cases of habitual constipation with very satisfactory results. We looked upon the constipation in these cases as a result of debility, inaction, or perhaps partial paralysis of the muscular coat of the intestines. By restoring vigor to the muscular tissue we reinstated the function of the bowel.

All those who have made nervous disease a subject of special study, are inclined to enlarge its boundaries, and to include the nervous pathology in many of those diseases considered as purely zymotic. But it is evident that blood poison operates not unfrequently, (and perhaps always) first upon the nervous system. And it is the ganglionic system which is especially affected by these poisons. The elimination of the poison is a natural process, but its rapidity must depend very much upon the integrity of the nervous centers which govern secretion. It is from looking at the subject in this light, that we were first enabled to recognize the philosophical nature of the quinine treatment in typhus. This theoretical opinion has since been confirmed by actual observation.

Now there is no great difference in the actions of quinine and strychnia. Both of them are stimulants directed especially to the ganglionic system; both are, *therefore*, anti-periodics, and they differ only in relative activity. Cases of typhoid fever occurring under the care of Prof. Rochester at the Buffalo Hospital of the Sisters of Charity during the past winter, which were in that low, sinking condition, verging toward coma, and marked by great debility of those organs depending on the ganglionic system for life, were greatly benefited, and apparently saved by the administration of strychnia.

We have not space to pursue this matter further, but we express our conviction that strychnia is a remedy of application almost as universal as brandy or quinine. In another number it is our intention to write more fully upon it.

*Oil and Albumen.*—The Monthly Journal of Medical Science, (Edinburgh,) has an article on the immunity of those engaged in wool-combing, and other greasy manufactures, from tubercle. It seems that consumption is very rare among them. Much of the oil which saturates their clothing is absorbed, and if the theory of Dr. Bennett be true, it furnishes the nuclei in cell formation, and prevents that excess of albumen which is asserted to be the principal or proximate cause of tuberculosis.

An ancient professional friend in Northern New York, where consumption is very frequent, and forms an enormous item in the bills of mortality, has been for some time engaged in experiment upon the inunction of cod liver oil in phthisis. What results he has obtained we do not know.

We translate from the "Moniteur des Hopitaux," the following account of an instrument for holding a sponge in making local applications to the pharynx; presented to the Societe de Chirurgie of Paris, at their sitting of the fourteenth of December 1853.

"*Porte éponge.*—At the commencement of the sitting, M. Robert presented in the name of Dr. C. J. Adams, secretary of the Academy of Medicine of New York, a whale-bone *porte éponge*, of three branches; which was made by M. Charriere, the son. This instrument was first invented by Dr. Buck, the Vice-president of the New York Academy, who constructed it with two branches.

"The design of this instrument is to permit the changing of the sponge which has served to cauterize one patient, when it is wished to cauterize another, without having recourse to the tedious process of binding on a new one, which is necessary whenever we wish to change the sponge in the ordinary instrument.

"One of the branches of the new instrument is furnished with a catch, over which can be passed a constricting ring, held by the aid of a bayonet notch. When the ring has reached the catch, a demi-movement of rotation is executed. We can then no longer pull off the sponge, and it is found to be immovably fixed."

Judging from the wood-cut, we should think this apparatus a little too complicated for so simple a purpose. The instrument which has been for some time in use, has but two branches, brought together by a simple constricting ring, without any catch. It is made of steel, and answers its purpose very well.

Either instrument is much better than the filthy habit of using the same sponge repeatedly.

*Union and Harmony.*—Judging from the objurgations of the Dublin Medical Press and its London correspondent, we should infer a very unpleasant state of things in the British medical world.

By some hocus-pocus which these writers do not make very clear, the ignorant, but designing men have succeeded in occupying all the good places, to the great detriment of honest worth and genuine talent. If we are to believe these authorities, every man of eminence in medicine or surgery, particularly those connected with the Royal Colleges and Hospitals, is an egregious rascal; while all the real talent and honesty is forced into chemists' shops, or into the poorer employment of surgeons in emigrant ships.

Everything is wrong there. Quacks are getting rich, and honest men grow lean and threadbare. They refer (*credat Judæus*) to America as the paradise of legitimate medicine.

We are sorry to learn so much evil of our transatlantic brethren. We can hardly believe that their sufferings are so intolerable. Perhaps when these writers, in the natural course of events, have themselves become "old fogies," and got good places in the Hospitals, they will feel better about it. Nothing makes a man so charitable to his neighbors as his own success. It is astonishing how good fortune, large fees, and many of them, do away with the evils of quackery, and dispose the happy recipient to lean back in his easy chair, stroke his abdomen, and say "*I* am very comfortable! what is the use of keeping up a continual disturbance about quackery?"

We believe there is a deal of exaggeration about quackery. We suppose that very few quacks attain to any great success. Now and then a pill-inventor may build up a fortune, but, so far as our own acquaintance extends, we have found that the ignoble herd of homœopaths, hydropaths, eclectics, and their brother humbugs, though eternally boasting of the amount of their business, never seem to realize from it all. For a few years they keep their heads above water by display at the expense of their creditors, and then sink to oblivion and poverty.

Saying nothing about the loss of caste, of social standing, and self-respect, involved in the practice of quackery, it has no material advantages to compensate, no dollar-and-cent charms to comfort one in his degradation.

We write this because we fancy that a great error has been committed by the medical press in this regard. It has uniformly held up the dishonors of of quackery as sure to meet with a splendid pecuniary reward, while it has given the most dolorous pictures of the sufferings of the honest medical man, toiling on through a life of poverty, unrewarded save by the halcyon calm of his own conscience. This is all moonshine! Poor ourselves, and with such a proclivity to the dispersion of the dollars, that we expect to remain so, we still think the profession of medicine a paying and an honorable occupation, and while we think that its responsibilitis and cares are too severe for the morbidly sensitive or lachrymose man, it still affords as pleasant a route through "this vale of tears" as any other for the cheerful, courageous, and enterprising.

*Surgical.*—In the proceedings of the Surgical Society of Ireland, J. H. Power, M. D., reports a case of dislocation at the tarso-metatarsal articulation, an accident which must necessarily be very rare. It was caused by jumping from a wrecked ship to a rock upon the shore, some eighteen or twenty feet distant from the spot whence he sprang.

The diagnostic marks of the accident were shortening of the foot, measuring from the heel to the toe, the tibia and os calcis in their natural position; the sole of the foot was convex, on the dorsum of the foot was a firm elevation with a corresponding depression, and finally, the foot was twisted.

The dislocation was adjusted by making extension from the great toe, the leg being flexed, and the ankle held by an assistant. Extension was then made in the direction of the abnormal axis of the great toe, that is, downward and forward. The right hand was then pressed against the projecting tumor in the sole of the foot, while the left did the same upon the dorsum. A sudden change was then made in the direction of the extension to upward and forward, with the result of bringing the bones to place.

*Amputation at the Hip Joint.*—This was performed July 5th, by Prof.

Van Buren, at the New York Hospital, on a little girl of 9 years, who had been run over by a railroad car, producing a compound comminuted fracture of the thigh bone. The operation was performed by the antero-posterior flaps — but little blood was lost.

Patient died forty-six hours after injury.

*Lithotomy.*—We have three curious cases of lithotomy to record. The first we abstract from the Edinburgh Monthly, the others from within our own observation.

Dr. Dinsmore performed lithotomy, and extracted a stone two inches long, one and a half broad, and three-fourths of an inch thick. It was found to consist of phosphates, and contained in its center an oval cavity, in which was *a portion of woolen cloth*, the size of a filbert.

This patient, eleven years before, while stepping into a fishing-boat, slipped and fell backward upon the "thole-pin," which entered the perineum near the anus. An abscess formed, and urine flowed through the opening, which remained open for five months. This cloth was evidently carried into the bladder by the "thole-pin." The reader will at once notice the comparatively trivial effects of so dangerous a wound, and set it by the side of Ahu Budka's operation, recorded on a previous page.

Our second case occurred during our pupilage, the operation being performed by our preceptor. The calculus was a formidable *slate pencil*, two and a half or three inches long, and somewhat incrusted with phosphates. This pencil the patient, a boy of twelve, had succeeded in navigating up the urethra as far as the neck of the bladder, when the attending surgeon finished the job by crowding it through into the bladder, in the attempt to extract it with forceps. A small perineal section would have saved some trouble in this case.

The third case is a very ludicrous, and, but for the fortunate explanation which was at last afforded, would have been a very annoying one to the distinguished surgeon and his colleagues, who all seemed at the time to have made a very singular error in diagnosis. A mulatto subject was laid upon the table to illustrate the operation of lithotomy. The surgeon was about to introduce a stone above the pubis as is usual, but on passing a sound, he discovered one already in the bladder. Materia Medica was called in, and after some hesitation, verified the diagnosis. Physiology came next, and the opinion was unanimous, that by a fortunate accident, they had a real stone in the bladder. The students were next called on. The click sound could be heard throughout the theater, and the calculus could be driven from side to side of the bladder, and was felt distinctly by the whole class.

Some water was now injected into the bladder, the operation was skillfully performed, with perhaps a little unusual display of dexterity. The finger was thrust in immediately, when, with a very blank countenance, the surgeon declared that he could find no stone. One after another examined with the same result. Physiology grew excited, split down the whole length of the urethra, and ripped up the abdomen, searched the ureters, turned the bladder inside out, but no stone. Materia Medica plumed himself highly upon his first hesitation in diagnosis, and laughed at his brothers in affliction.

There the matter rested. To be so deceived was very mortifying to a surgeon who had often performed the operation on the living subject. It went far to destroy his confidence in his own powers of diagnosis. The next day, the demonstrator, who had been absent, afforded the following laughable explanation. The subject had lain for a week, completely frozen, and had only got well thawed upon the surface, when brought into theater. Ice from frozen urine still remained in the bladder, and this it was that had afforded the click sound, (which had been noticed as somewhat peculiar.) During the prolonged examination and the injection of the water, the ice had thawed, and of course, the *calculus* had disappeared.

There is only one moral to this history so far as we know. All lithotomists should be sure that their patients have not ice in their bladders before they operate.

*Encyclopædiacal Knowledge.*—We find the following "nut to crack," quoted in the Virginia Medical and Surgical Journal.

"It is all very fine to insist that the eye cannot be understood without a knowledge of optics, nor the circulation without hydraulics; that metaphysics have their use in leading us through the intricate functions of the nervous system, and the mysterious connections of mind and matter. It is a truth; and it is a truth also that the whole circle of the sciences is required to comprehend a single atom of matter; but the most solemn truth of all is, that the life of man is three score years and ten."

It is no mean question with the practical physician — he who wishes to know what he may use to the benefit of his patients — as to which of all the surgical books thrown out to the profession, are most necessary and useful to him. The depths of comparative anatomy, the mysteries of the cell, are subjects for the philosopher, rather than the physician. The latter may profit by the general deductions of the former, he may grasp the thought which governs these arcana, but he cannot become familiar with their detail, without sacrificing time which he can poorly spare. H.

*Pharmaceutical.*—Mr. A. I. Mathews, an enterprising and excellent druggist of this city, has sent us his annual catalogue, a pamphlet of some 120 pages, filled with a long array of drugs and medicines, pharmaceutical preparations, surgical instruments, etc. Among them all, we find no mention of any patent or secret remedies, neither are such to be found upon his shelves.

Accompanying the catalogue, we have received a suite of specimens of some new pharmaceutical products, prepared by himself, in very convenient form for administration.

These consist of fluid extracts, and of saccharated powders. The extracts are fourteen in number, one-half of them being officinal, and the remainder prepared from formulæ which have appeared in the American Journal of Pharmacy. They are extremely eligible and convenient for exhibition. They retain the characteristic odor of the plant in a marked degree. As an instance of convenience, we may notice the "Ext. Spigeliæ et Sennæ Fluid," of the U. S. Pharmacopeia. The dose of this active anthelmintic is only a fluid dram for a child two years old. Besides this we have the fluid extracts of Senna, Valerian, Hyosciamus, Serpentaria, Lobelia, Rhei et Sennæ, Buchu, Cubebs, Cinchona, Piper Niger, Gentian, Rhubard, and Sarsaparilla.

The saccharated powder is something entirely new to us. It is prepared by evaporating the tinctures of plants to dryness, with an equal weight of sugar. They are extremely neat in appearance, pleasant in exhibition, and, if we are to judge from odor and taste, active in medical potency. Hyosciamus, Aconite, Cinchona, Valerian, Carbonate of Iron and Manganese, Digitalis, and Sanguinaria, are thus put up, a solid dram of the powder being equal to a fluid dram of the tincture.

We are glad to notice such beautiful preparations by a Buffalo pharmaceutist, and do not see why they should not be generally adopted. H.

